# Beta-1,3 Oligoglucans Specifically Bind to Immune Receptor CD28 and May Enhance T Cell Activation

**DOI:** 10.3390/ijms22063124

**Published:** 2021-03-18

**Authors:** Jeffrey Comer, Molly Bassette, Riley Burghart, Mayme Loyd, Susumu Ishiguro, Ettayapuram Ramaprasad Azhagiya Singam, Ariela Vergara-Jaque, Ayaka Nakashima, Kengo Suzuki, Brian V. Geisbrecht, Masaaki Tamura

**Affiliations:** 1 Department of Anatomy and Physiology, Kansas State University, Manhattan, KS 66506, USA; Molly.Bassette@ucsf.edu (M.B.); rireann@ksu.edu (R.B.); mkloyd@ksu.edu (M.L.); isusumu@vet.k-state.edu (S.I.); eazhagiy@berkeley.edu (E.R.A.S.); arvergara@utalca.cl (A.V.-J.); 2 Molecular Graphics and Computation Facility, College of Chemistry, University of California, Berkeley, CA 94720, USA; 3 Center for Bioinformatics, Simulation and Modeling (CBSM), Faculty of Engineering, Universidad de Talca, Talca 3460000, Chile; 4 Euglena Co., Ltd., Tokyo 108-0014, Japan; nakashima@euglena.jp (A.N.); suzuki@euglena.jp (K.S.); 5 Department of Biochemistry and Molecular Biophysics, Kansas State University, Manhattan, KS 66506, USA; geisbrechtb@ksu.edu

**Keywords:** beta glucans, oligoglucans, oligomers, CD28, CD3, T cell activation, immune stimulation, molecular dynamics simulation, free energy calculation

## Abstract

Beta glucans are known to have immunomodulatory effects that mediated by a variety of mechanisms. In this article, we describe experiments and simulations suggesting that beta-1,3 glucans may promote activation of T cells by a previously unknown mechanism. First, we find that treatment of a T lymphoblast cell line with beta-1,3 oligoglucan significantly increases mRNA levels of T cell activation-associated cytokines, especially in the presence of the agonistic anti-CD3 antibody. This immunostimulatory activity was observed in the absence of dectin-1, a known receptor for beta-1,3 glucans. To clarify the molecular mechanism underlying this activity, we performed a series of molecular dynamics simulations and free-energy calculations to explore the interaction of beta-1,3 oligoglucans with potential immune receptors. While the simulations reveal little association between beta-1,3 oligoglucan and the immune receptor CD3, we find that beta-1,3 oligoglucans bind to CD28 near the region identified as the binding site for its natural ligands CD80 and CD86. Using a rigorous absolute binding free-energy technique, we calculate a dissociation constant in the low millimolar range for binding of 8-mer beta-1,3 oligoglucan to this site on CD28. The simulations show this binding to be specific, as no such association is computed for alpha-1,4 oligoglucan. This study suggests that beta-1,3 glucans bind to CD28 and may stimulate T cell activation collaboratively with T cell receptor activation, thereby stimulating immune function.

## 1. Introduction

β-1,3 glucans are polymers of glucose characterized by β-1→3 glycosidic linkages. Similar polysaccharides, which are typically produced by fungi, plants, and microorganisms [1] may be referred to as curdlan [2,3], laminarin [4], lentinan [5], pachyman [6], paramylon [3], pleuran [7], or zymosan [8], depending on the species from which they originate and their specific composition. In addition to 1→3 linkages, these substances often contain other carbohydrate motifs, notably 1→6 branches. Given their association with fungi and microorganisms, many animals, including humans, exhibit strong immune responses to β-1,3 glucans [1,9,10]. Therefore, β-1,3 glucans can cause inflammation [11]; however, paradoxically, foods rich in β-1,3 have shown positive effects on health [12]. Cereal glucans, such as oat glucans, which contain 1→3 and 1→4 linkages, have been established as promoters of cardiovascular health [13,14]. Moreover, the immunostimulatory effects of β-1,3 have been widely studied for cancer treatment [1,15,16]. Anticancer activities of extracts of some single-celled algae may also be linked to β-1,3 glucans [17].

Linear β-1,3 glucans of up to 20 monomers are soluble in water [18], while reduced length and the presence of 1→6 linkages increases water solubility [19]. Soluble oligomers can show greater pharmacological activity than large polymers; for instance, oligosaccharides (mostly 2-mer and 3-mer, having molecular masses of 0.36 to 0.54 kDa) formed from glucans were shown to possess greater antidiabetic activity than crude glucans (30–50 kDa) [20].

Complement receptor 3 (CR3) [21], dectin-1 [22], and TLR-2 [23] have been implicated in the recognition of β-1,3 glucans by the innate immune system. The β-1,3 glucan zymosan, in particular, has been shown to activate TLR-2 [23]. However, other immune system receptors may also interact in important ways with β-1,3 glucans [18]. In this article, we describe cell culture experiments demonstrating that β-1,3 oligoglucans induce expression of cytokines associated with T lymphocyte activation. We apply molecular dynamics simulations to explore the possible molecular mechanisms for this biological activity, which suggest a role of CD28 in the observed immunostimulatory effect of β-1,3 oligoglucans. Here, we report for the first time that β-1,3 glucan can specifically bind to CD28 at the region identified as the binding site for its natural ligands CD80 and CD86, potentially revealing a novel mechanism for immunostimulation by β-1,3 glucans.

## 2. Results and Discussion

### 2.1. β-1,3 Oligoglucan Treatment Induced Expression of T Lymphoblasts Activation-Associated Cytokines

To evaluate the effect of β-1,3 glucans on the functional stimulation of human T lymphocytes, we measured expression of selected cytokines in a model cell type (Jurkat T lymphoblasts) treated with 5-mer β-1,3 oligomers. It should be noted that Jurkat cells do not express dectin-1, a well-known receptor for β-1,3 glucans, [24,25], so any observed activity must be due to a mechanism not involving this receptor. To determine the specificity for β-1,3 glucans, the experiments were repeated with 5-mer α-1,4 oligoglucans. Injection of phosphate-buffered saline (PBS) served as a negative control, while injection of a combination of anti-CD3 and anti-CD28 antibodies was a positive control for T cell activation. Gene expression of interleukin-2 (IL-2), interferon γ (IFNγ), granzyme B (GZMB), and tumor necrosis factor (TNFα), in Jurkat cells was measured by real-time PCR.

**Figure 1:**
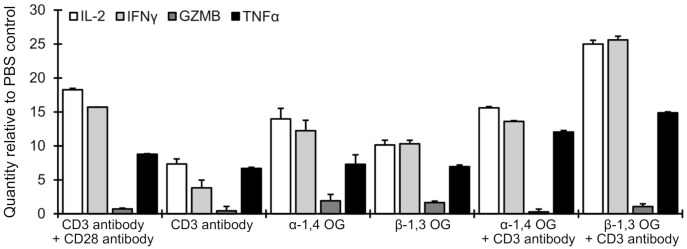
Effect of oligoglucans (OG) on expression of selected effector molecules in Jurkat cells as evaluated by real-time PCR 24 h after treatment with 10 µg/mL OG. Values are normalized to the PBS control for that effector molecule; hence, a value of 1.0 indicates no difference from the PBS control. Results for treatment with a combination of anti-CD3 and anti-CD28 antibodies are included as a positive control for T cell activation. All experiments were performed in triplicate with error bars representing standard errors.

The results of RNA extraction and real-time PCR performed 24 h after after treatment are shown in Figure 1, plotted relative to the PBS control. Three of the tested cytokines (IL-2, TNFα, and IFNγ), but not the effector molecule GZMB, were upregulated 24 h after treatment with α-1,4 or β-1,3 oligoglucans. Furthermore, expression of IL-2 and IFNγ was significantly higher in the presence of the oligoglucans than in the presence of the anti-CD3 antibody, which was used as a positive control. A combination of β-1,3 oligoglucan with the anti-CD3 antibody led to the greatest IL-2 and IFNγ expression, while a combination of α-1,4 oligoglucan with the anti-CD3 antibody gave results similar to α-1,4 oligoglucan alone for these cytokines. These results suggest that β-1,3 oligoglucan treatment stimulates activation of T lymphocytes by interaction with CD28 on the T cell plasma membrane and this stimulation is more effective when it is accompanied by CD3 activation. On the other hand, minimal GZMB induction by β-1,3 oligoglucan suggests that this CD28-dependent T cell activation is partial and insufficient to induce the development of effector T cells. In support of this notion, neither 5-mer α-1,4 nor 5-mer β-1,3 glucans stimulated growth of T lymphoblasts in either in the presence or absence of agonistic anti-CD3 and anti-CD28 antibodies (see Figure S1 of the Supporting Information).

### 2.2. Interaction of β-1,3 Oligoglucans with Immune Receptors.

**Figure 2:**
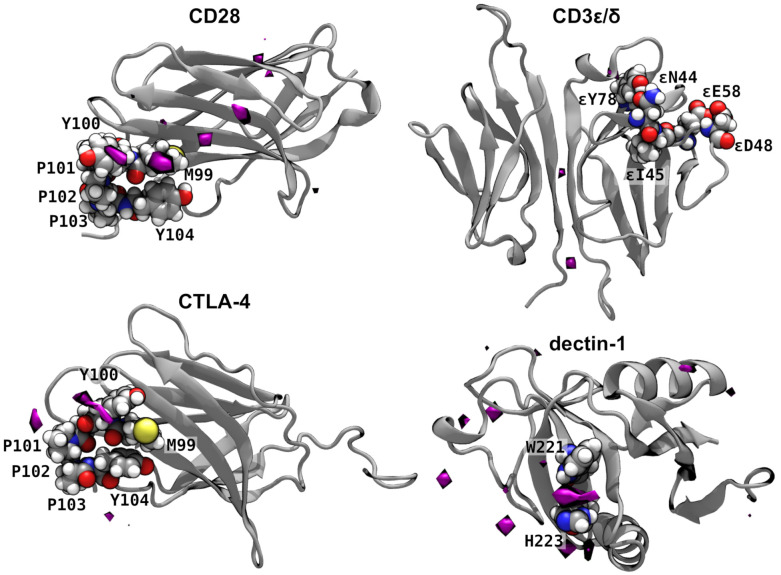
Interaction of immune receptors with 8-mer β-1,3 oligoglucans. The purple surfaces enclose regions where the concentration of glucan monomers was greater than 30 times the ambient concentration. Notable residues are shown as atomic spheres (H, white; C, gray; N, blue; O, red; S, yellow). The remaining parts of the proteins are shown by gray secondary structure representations.

To understand the experimental results shown in Figure 1, we performed atomistic molecular dynamics simulations to explore the interaction of β-1,3 oligoglucans with proteins involved in T cell activation. Binding of the glucans to a functionally relevant site of CD3 or CD28 might be able to cause the observed T cell activation. Therefore, we performed simulations of CD28 [26] and the CD3 є/δ dimer [27] in solution containing randomly placed 8-mer β-1,3 oligoglucan molecules. For robustness, these simulations were performed in duplicate for each protein, with different initial placement of the oligoglucan molecules. The spatial distribution of glucose units of these molecules was averaged over the two simulations; regions where the oligoglucans had a high propensity to linger are shown in Figure 2. We observed that β-1,3 oligoglucan molecules reversibly associate with CD28 near residue Met99. Consequently, Figure 2 reveals an exceptionally high density of β-1,3 glucan in this region. The region near Met99 is particularly important for the function of CD28, as it coincides with the highly conserved MYPPPY loop, which has been suggested as the binding site between CD28 and its ligands, CD80 and CD86 (also known as B7-1 and B7-2, respectively) [28], as well as the binding site of an agonistic anti-CD28 antibody [26]. On the other hand, little association between β-1,3 oligoglucan and the CD3 є/δ dimer was observed, apart from slightly enhanced glucan density at the interface between the CD3є and CD3δ subunits (Figure 2). Importantly, no appreciable association was observed near the ligand binding site, comprising residues 44, 45, 48, 58, and 78 of the chain [27].

CTLA-4 possesses considerable structural similarity with CD28, having the same MYPPPY loop, and binding the same ligands (CD80 and CD86). However, while CD28 is responsible for T cell simulation, CTLA-4 downregulates immune responses. [29] As shown in Figure 2, we also observed some association of β-1,3 oligoglucan near the MYPPPY loop; however, the propensity to associate and remain there appeared much weaker than for CD28. The reason for this weaker association seems to be due to the fact that the -sheet adjacent to the MYPPPY loop in CTLA-4 does not fit snugly against residues Met99 and Tyr100, but is displaced toward the center of the protein; hence, the channel where β-1,3 glucan binds in CD28 is absent in CTLA-4.

Dectin-1 is a well known as a pattern-recognition immune receptor for glucans containing β-1,3 and β-1,6 linkages; however, it cannot be responsible for the β-1,3 oligoglucan activity observed in the cell culture experiments since the model cell line (Jurkat) does not express dectin-1 [24,25]. For comparison, we considered the interactions of the β-1,3 oligoglucan with dectin-1. As expected, we observed association of the β-1,3 oligoglucan with dectin-1 (Figure 2), particularly between the side chains of residues Trp221 and His223, which have been implicated in β-1,3 glucan binding [30].

These simulations suggest that dectin-1 and CD28 have similar affinities for β-1,3 oligoglucan. The ratio, *M*, of the maximum glucan concentration to its ambient concentration is 140 for dectin-1 and 100 for CD28. The volumes of the high density (30 times ambient concentration) regions are also similar: 200 and 120 Å^3^ for dectin-1 and CD28, respectively. In contrast, CTLA-4 and CD3 є/δ show comparably weaker binding of the 8-mer β-1,3 oligoglucans, with *M* values of 57 and 78 and volumes of 71 and 24 Å^3^. Altogether, these simulations that β-1,3 oligoglucans may bind with reasonably high affinity to CD28 near its ligand binding site.

### 2.3. Specificity of β-1,3 Interaction with CD28.

The determine the specificity of the association between β-1,3 oligoglucans and CD28, we performed simulations similar to those described in the last section including multiple 5-mers of either α-1,4 or β-1,3 oligoglucans. Again, these oligoglucan molecules diffused around CD28, occasionally making contact with different regions of the protein. The unbiased simulations were performed in triplicate, with each of the three replicas having different initial oligoglucan positions and orientations. As in the simulations described in Figure 2 for 8-mer β-1,3 oligoglucans, the 5-mer β-1,3 molecules were observed to intermittently bind for a relatively long period (>50 ns) to a region of CD28 near residue 99. Similar binding events were not observed for 5-mer α-1,4 oligoglucan.

**Figure 3:**
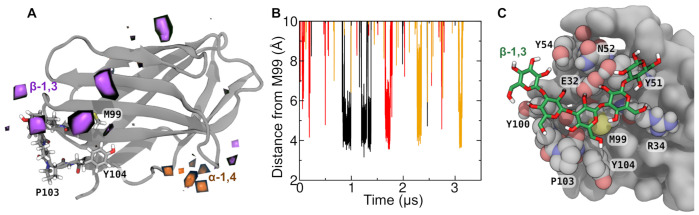
Binding of β-1,3 oligoglucan to the immune receptor CD28. (**A**) Regions of high oligoglucan density in unbiased molecular dynamics simulations containing many 5-mer oligoglucan molecules (-1,4 or β-1,3). Regions where the average density of oligoglucan monomers is 8 times their ambient density are highlighted in orange (-1,4) or purple (-1,3). The MYPPPY loop (residues 99–104), which makes up part of the ligand binding site of CD28, is shown explicitly, while the remainder of CD28 is shown in a gray secondary structure representation. (**B**) Distance between three selected β-1,3 oligomer monomers and residue Met99 during in two replicates of the simulation. We selected curves where there is contact (center-of-mass separation <5 Å) between the glucose moiety and sulfur atom of Met99 continuously for >30 ns. (**C**) Exemplary simulation frame where a 5-mer β-1,3 oligoglucan is bound to CD28. Residues of CD28 making contact with the oligoglucan are shown as atomic spheres (carbon in gray), while the oligoglucan is shown in a bonds representation (carbon in green).

The statistics of the binding events observed in the simulation are summarized in Figure 3A, which highlights regions on CD28 where the density of the monomers of the 5-mer oligoglucan is >8 times the density of these monomers far from the protein. Clear high density regions of 5-mer β-1,3 oligoglucan are seen near the MYPPPY motif, particularly near residues Met99 and Tyr100. No comparably large regions of high density are seen for the 5-mer α-1,4 oligoglucan, and the smaller regions of high α-1,4 oligoglucan density that do appear are not near the ligand-binding site.

Analysis of the simulation trajectories show several independent binding and unbinding events, examples of which are shown in Figure 3B. These events involve three distinct β-1,3 oligoglucan molecules and two of the three simulation replicates. The black and red curves in Figure 3B show binding events from two different oligoglucan molecules in the first replicate. The black curve shows that one of the oligoglucan molecules lingers near Met99 from *t* = 0.84 µs to 0.99 µ. During this time, the distance between the center of mass of the central monomer of the oligoglucan and the sulfur atom of Met99 is usually <5 Å. This same oligoglucan molecule binds again in two consecutive events (1.19≤t≤1.30 µs and 1.32≤t≤1.38 µs), separated by a short period where the distance between the central monomer and Met99 exceeds 27 Å. In the same simulation, after dissociation of this first molecule, a different (albeit chemically identical) oligoglucan molecule associated near Met99 during 1.66≤t≤1.75 µs (red curve). The orange curve in Figure 3B shows binding of a β-1,3 oligoglucan to the same site in an independent replicate during times 2.26≤t≤2.35 µs.

During the observed binding events, the β-1,3 oligoglucan molecule inserted itself into a channel on the surface of CD28. A representative configuration of the CD28:β-1,3 oligoglucan complex during these binding events is shown in Figure 3C. Typically, the pyranose rings of the glucan adopted a relatively flat conformation, lying on top of residues Tyr51, Met99, and Tyr100, which make up the bottom of the channel. The β-1,3 oligoglucan forms H-bonds with groups on the sides of the channel. One or two H-bonds were formed between Glu32 and hydroxyl groups at the 2 positions of the glucan in nearly all simulation frames where the oligoglucan appears bound. H-bonds were also intermittently formed with the backbone carbonyls of Met99, Pro103 and Ser30; the side chain amide of Asn52; the guanidinium groups of Arg31 and Arg34; and the side chain hydroxyls of Tyr51 and Tyr54. The bound conformation of the β-1,3 oligoglucan seemed to be further reinforced by intramolecular H bonds between position-4 hydroxyl groups and the O5 atoms of the pyranose ring of the previous glucose monomer. Due its distinct chemical structure, the α-1,4 oligoglucan appears unable to adopt a similar flat conformation where it can make hydrophobic contacts with the bottom of the channel and H-bonds with residues on the channel sides.

### 2.4. Three-Dimensional Free Energy Calculations

**Figure 4:**
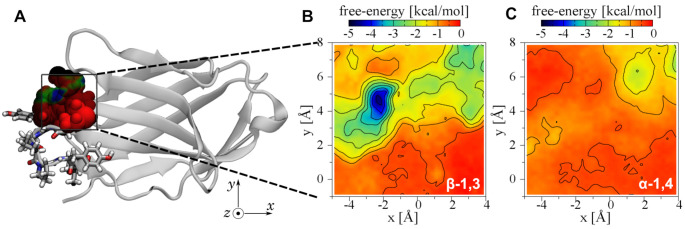
Three-dimensional free-energy map for 5-mer oligoglucans in the vicinity of Met99 of CD28. The free energy is calculated as a function of the position of the central glucose monomer of a 5-mer β-1,3 oligoglucan relative to the center of mass of residue 99 of CD28. (**A**) Location of the mapped region of CD28. Atoms within the mapped region are shown as spheres colored according to the projected minimum free energy. (**B**,**C**) Projections of the three-dimensional free-energy map showing the minimum free energy of each line (x,y) along the *z*-axis.

After identifying the region that appeared to serve as a binding site for β-1,3 oligoglucans, we sought to quantify the associated interaction. We mapped the free energy of 5-mer β-1,3 and α-1,4 oligoglucans in three dimensions in a region near the residue Met99. The displacement vector of the center of mass of the central monomer of the 5-mer oligoglucan relative to the center of mass of Met99 served as the three transition coordinates. Figure 4 displays projections of the three-dimensional free energy maps. Figure 4B shows that, for the 5-mer β-1,3 oligoglucan, a highly favorable free energy exists near residues Met99 and Tyr100 of the MYPPPY loop, as well as residues 32 to 34 of the adjacent strand. On the other hand, for the 5-mer α-1,4 oligoglucan, no such favorable region is visible in Figure 4C. Hence, from Figure 4, we infer that CD28 possesses a binding site selective for β-1,3 oligoglucan.

### 2.5. Calculation of the Standard Binding Free Energy

Finally, we applied a computational protocol [31,32,33] to rigorously estimate the standard free energy for binding of 8-mer β-1,3 oligoglucan to the site on CD28 identified in previous sections. The resulting value was $\Delta G^{{}^{\circ}}=-2.56\pm 0.48$ kcal/mol, which corresponds to a dissociation constant ($K_{d}$) in the range 7–34 mmol/L. Although the affinity of these glucans for CD28 is relatively low, it may be sufficient for the biological effect observed in the experiments. It should be noted that this $K_{d}$ value is comparable to the IC_50_ value of dectin-1 for the same 8-mer oligoglucan (1.1 mmol/L) [34]. Furthermore, the affinity may vary depending on the length and the presence of 1→6 linkages. Notably, dectin-1 has a much greater affinity for β-1,3 oligoglucans including 1→6 linkages and β-1,3 glucans from natural products than their linear congeners [34].

## 3. Conclusions

The molecular dynamics simulations reveal that 5-mer and 8-mer β-1,3 oligoglucans have a propensity to bind near the same region of CD28 associated with binding of its natural ligands CD80 and CD86. This binding was seen to be selective, because the affinity of α-1,4 oligoglucan for the same site was much lower. Indeed, the affinity of linear β-1,3 oligoglucan for CD28 was similar to that for dectin-1, which is well-established as a receptor for β-1,3 oligoglucan. To our knowledge, this is the first demonstration of the possibility of a specific interaction between β-1,3 oligoglucans and CD28. The validity of the simulation approach is supported by the qualitatively reasonable results obtained for dectin-1. Consistent with the results of the simulations, we found that 5-mer β-1,3 oligoglucan induced expression of T lymphocyte activation-associated cytokines in T lymphoblasts in cell culture. The present study strongly suggests that binding of β-1,3 oligoglucan to CD28 present on the T cell plasma membrane functionally stimulates T cell activation in collaboration with CD3 activation. Since expression of the β-1,3 glucan receptor dectin-1 is negligible on Jurkat cells [24], a dectin-1-mediated mechanism is unlikely to explain our results. This work provides evidence that CD28 plays a role in the immunostimulatory effects of glucans, which may be relevant in the glucan-induced anticancer immunity observed in preclinical studies.

## 4. Materials and Methods

Maltopentaose (5-mer α-1,4 oligoglucan) was purchased from Santa Cruz Biotechnology (Dallas, TX, USA), while laminaripentaose (5-mer β-1,3 oligoglucan) was purchased from Megazyme (Bray, Ireland). The Jurkat human lymphoblast cell line (TIB-152) was from American Type Culture Collection (ATCC, Manassas, VA, USA). RPMI 1640 was obtained from Mediatech, Inc. (Manassas, VA, USA). Fetal bovine serum (FBS) was from EQUITECH-BIO Inc. (Kerrville, TX, USA). Penicillin-streptomycin stock solution was from Lonza Rockland, Inc. (Allendale, NJ, USA). The anti-CD3 and anti-CD28 antibodies were purchased from BioLegend (San Diego, CA, USA).

### 4.1. Cell Culture

The Jurkat cells were cultured at 37 °C in a humidified air atmosphere containing 5% CO_2_ using RPMI1640 medium supplemented with 10% *v/v* FBS and 1% *v/v* penicillin-streptomycin. The Jurkat cells (human T lymphoblasts, 1000 cells/well) were seeded into a 96 well plate with 100 µL of growth medium. After 24 h, the cells were treated with either 5-mer α-1,4 or β-1,3 oligoglucan (10 or 100 µM). Cell growth was evaluated from 48 through 96 h after treatment using 3-(4,5-dimethylthiazol-2-yl)-2,5-diphenyltetrazolium bromide (MTT) assay as previously described [35,36]. PBS was used as the negative control. In addition, ^3^H-thymidine incorporation was evaluated under previously described [37] conditions.

### 4.2. Analysis of Cytokine Expression

Gene expression of interleukin-2 (IL-2), tumor necrosis factor α (TNFα), interferon (IFNγ), and granzyme B (GZMB) in α-1,4- and β-1,3-treated Jurkat cells was determined by reverse-transcription quantitative polymerase chain reaction (RT-qPCR). Jurkat cells (1×10^5^ cells/well) were seeded into a 12-well plate. After 24 h, cells were treated with 10–100 M of either 5-mer α-1,4 or 5-mer β-1,3 oligoglucan. For a positive control of T-cell activation, cells were treated with a combination of anti-CD3 (1 µg/mL) and anti-CD28 (5 µg/mL) antibodies. Treatment with anti-CD3 antibody alone was also at 1 µg/mL. Total RNA was extracted and purified at 24 and 48 h after treatment using TRIzol reagent (Thermo Fisher Scientific, Waltham, MA, USA). One step RT-qPCR was carried out using the iTaq Universal SYBR Green One-Step Kit (Bio-Rad, Hercules, CA, USA), and the reactions were conducted on the StepOnePlus Real-Time PCR System (Applied Biosystems, Waltham, MA, USA). The qPCR was performed as follows: 45 cycles of 15 s at 95 °C, and 60 s at 60 °C. The results were quantified by the comparative $C_{T}$ method [38]. The sequences of primers used are described in Table 1. The experiments were performed in triplicate.

### 4.3. Molecular Models

Models of CD28 [26] CTLA4, [28], and the CD3 є/δ dimer [27] were constructed based on existing experimental structures of their human forms (PDB IDs: 1YJD, 1AH1, and 1XIW). For dectin-1, we used the the murine form, which is the only one available (PDB ID: 2CL8) [39]. The formation of a dimer and the positioning of a 3-mer β-1,3 at the dimer interface in this dectin-1 structure are not thought to be biologically relevant; hence, we extracted only a single dectin-1 monomer for the simulations. The proteins were prepared for simulation using the CHARMM-GUI server [40]. Disulfide bridges were added (residues 22:94 and 48:68 of CD28; residues 21:94 and 48:68 of CTLA4, residues 16:52 and 28:77 of CD3 є/δ; and residues 119:130, 147:240, and 219:232 of dectin-1) in accord with the structural analyses [27]. In the simulations, proteins were represented using the CHARMM36m force field [41,42,43,44].

Five-mer and 8-mer α-1,4 and β-1,3 oligoglucans were created by replicating -d and -d glucose units and linking them with 1→4 or 1→3 glycosidic linkages. Interatomic interactions involving polysaccharides were defined according to the CHARMM36 carbohydrate force field [45,46]. For each simulation, the oligoglucan models were combined with the protein model as described below. For each 5-mer oligoglucan system, ≈5400 water molecules were added to form a simulation box with average equilibrated dimensions of 51 × 62 × 57 Å^3^. The longer 8-mer oligoglucans required larger boxes to prevent the oligoglucans from simultaneously contacting multiple periodic images of the protein; hence, these systems contained ≈19000 water molecules and had dimensions of 81 × 90 × 83 Å^3^. In all cases, Na^+^ and Cl^−^ ions were added to obtain a salt concentration of ≈150 mmol/L. A few ions were also added to neutralize the systems, according the total charge of the protein models.

### 4.4. Molecular Dynamics Simulations

All molecular dynamics simulations were performed using the program NAMD. Consistent with the CHARMM force field framework, water molecules [47] and covalent bonds involving hydrogen [48] were kept rigid. The equations of motion were integrated with a 4 fs timestep, made possible by mass repartitioning of the protein and oligoglucan molecules [49]. The masses of non-water hydrogen atoms were scaled by a factor of three and the masses of atoms bonded to them were reduced to compensate. Full electrostatic interactions were efficiently implemented by the particle-mesh Ewald algorithm [50] (grid spacing ≤1.2 Å). Lennard-Jones and direct electrostatic forces were truncated at a distance of 9 Å, with a smooth approach beginning at 8 Å. In all cases, the Langevin thermostat and Langevin piston barostat [51] algorithms were used to maintain a temperature of 310 K and a pressure of 1.01325 bar. Specific Lennard-Jones parameters between Na^+^ and Cl^−^ ions [52] and between Na^+^ carboxylate oxygen atoms [53] were included. For convenience, select atoms in the core of the protein were restrained to their initial positions to prevent diffusion or reorientation of CD28. Because these restraints act only on the protein’s center of mass, they do not affect calculation of the binding thermodynamics.

### 4.5. Unbiased Simulations

Simulations were performed in which twelve 5-mer or eight 8-mer oligoglucan oligoglucans were initially placed randomly around the proteins with no oligoglucan atom nearer than 8 Å from the protein using Packmol [54]. Simulations with 8-mer β-1,3 oligoglucans were performed in duplicate (distinct initial conditions) for CD28, CD3 є/δ, CTLA-4, and dectin-1, totaling 3.2 s for each protein. For each protein/oligoglucan combination, after aligning the protein in each simulation frame, the mean density of glucose units was calculated on a three-dimensional grid with a spacing of 2.0 Å. For CD28, an additional set of simulations were performed in triplicate (beginning from three distinct initial oligoglucan arrangements) with 5-mer α-1,4 or β-1,3 oligoglucans, totaling 10.6 s for each oligoglucan.

### 4.6. Three-Dimensional Free Energy Calculations

Free energies were calculated using the adaptive biasing force (ABF) method [55,56]. The protein was oriented so that displacement along the *z*-axis would extract the oligoglucan from the binding site observed in the unbiased simulations (see Figure 4). To determine how the free energy varied with the location of the oligoglucan molecule, we first performed an ABF calculation in three dimensions, using the *x*, *y*, and *z* coordinates of the center of mass of the central monomer of the oligoglucan molecule relative to the center of mass of Met99 as the transition coordinates. The domain of each coordinate was x∈[−5,4] Å, y∈[−1,8] Å, and z∈[0,18] Å, with a grid spacing of 0.25 Å along each direction. Two independent simulations with different initial conditions were performed for each oligoglucan (-1,4 or β-1,3), and the results were combined to produce Figure 4. The simulation time totaled 3.1 µs for each oligoglucan.

### 4.7. Calculation of the Standard Binding Free Energy

The standard binding free energy for an 8-mer β-1,3 oligoglucan molecule complexed with CD28 was calculated by ABF using the Binding Free Energy Estimator (BFEE) plugin [33] of VMD [57]. To make the computation tractable, this plugin implements an 8-stage simulation protocol [32] that calculates the free energy for artificially restraining the ligand to the bound reference structure (stage 1–6), the standard free energy for dissociating the restrained ligand from the protein (stage 7), and the free energy for removing the restraints from the restrained ligand in solution (stage 8 plus analytical computations). Because the end points correctly represent an unrestrained complex and unrestrained free ligand and free protein, the sum over these free energies gives a rigorous absolute binding free energy. The bound reference structure was extracted from the trajectories of the unbiased CD28 simulations described above, using the non-rigorous MM-GBSA (molecular mechanics/generalized Born/surface area) method [58] to select an appropriate bound structure. The unbiased trajectories were processed to remove water, Na^+^ and Cl^−^ ions, and all but one oligoglucan. The MM-GBSA estimates of the CD28:oligoglucan binding free energies ($\Delta G^{\mathrm{MM}}-{\mathrm{GBSA}}_{\mathrm{bind}}$) were then estimated for each trajectory frame. As these were single-point estimates, conformational entropy was not calculated. The frame with the lowest $\Delta G^{\mathrm{MM}}-{\mathrm{GBSA}}_{\mathrm{bind}}$ was selected as the initial structure for series of BFEE calculations.

The simulation times for each stage are listed in Table 2. Consistent with the other simulations described in this work, a 4 fs timestep was used. Stage 7 is the calculation where it is most difficult to obtain sufficient sampling; hence, two independent calculations were performed (with different initial velocities) for stage 7. To calculate the uncertainty, the data for each stage was partitioned into two segments. For stage 7, these two segments were obtained from the two independent simulations, while, for the other stages, the two segments corresponded to the first and second halves of a single simulation (see the supporting information of ref. [59]). We applied the BFEE plugin separately for the two segments and calculated the uncertainties as the maximum deviation between the values from all data and the values from the two segments.

## Figures and Tables

**Table 1:** Primers used for RT-qPCR.

Primer		Sequence	Size
Human	Forward (5${}^{\prime }$–3${}^{\prime }$)	ATGAGACAGCAACCATTGTAGAATTT	87 bp
IL-2	Reverse (5${}^{\prime }$–3${}^{\prime }$)	CACTTAATTATCAAGTCAGTGTTGAGATGA	

Human	Forward (5${}^{\prime }$–3${}^{\prime }$)	GCCAGAATGCTGCAGGACTT	63 bp
TNFα	Reverse (5${}^{\prime }$–3${}^{\prime }$)	GGCCTAAGGTCCACTTGTGTCA	

Human	Forward (5${}^{\prime }$–3${}^{\prime }$)	AGGGAAGCGAAAAAGGAGTCA	64 bp
IFNγ	Reverse (5${}^{\prime }$–3${}^{\prime }$)	GGACAACCATTACTGGGATGCT	

Human	Forward (5${}^{\prime }$–3${}^{\prime }$)	TGCAGGAAGATCGAAAGTGCG	180 bp
GZMB	Reverse (5${}^{\prime }$–3${}^{\prime }$)	GAGGCATGCCATTGTTTCGTC	

18S	Forward (5${}^{\prime }$–3${}^{\prime }$)	GAGGTTCGAAGACGATCAGA	315 bp
	Reverse (5${}^{\prime }$–3${}^{\prime }$)	TCGCTCCACCAACTAAGAAC	

**Table 2:** Free-energy values and simulation times for each stage of the rigorous absolute binding free energy calculation [32]. ${}^{†}$ Stage 9 is computed analytically and requires no simulation.

Stage	System	Free-Energy	Free Energy	Sim. Time
		Term	(kcal/mol)	(ns)
1	protein–ligand	$\Delta G_{\mathrm{conform}}$	−6.79±0.08	200
2	protein–ligand	$\Delta G_{\Theta }$	−0.46±0.05	200
3	protein–ligand	$\Delta G_{\Phi }$	−0.48±0.08	170
4	protein–ligand	$\Delta G_{\Psi }$	−0.37±0.05	160
5	protein–ligand	$\Delta G_{\theta }$	−0.20±0.07	120
6	protein–ligand	$\Delta G_{\phi }$	−0.10±0.01	100
7	protein–ligand	$-k_{B}T\ln\left(S^{*}I^{*}C^{{}^{\circ}}\right)$	−8.46±0.33	1190
8	ligand only	$\Delta G_{\mathrm{conform}}^{\mathrm{unbound}}$	+7.49±0.09	240
9	ligand only	$\Delta G_{\Theta \Phi \Psi }^{\mathrm{unbound}}$	+6.80±0.00	0 ${}^{†}$
total	–	$\Delta G^{{}^{\circ}}$	−2.46±0.48	2380

## Data Availability

Selected simulation input parameters and output data are openly available in Zenodo (DOI: 10.5281/zenodo.4609401). Other data is available upon request.

## Supplementary Materials

The following are available online at https://www.mdpi.com/1422-0067/22/6/3124/s1, Figure S1: Cell proliferation assays.
