# Supplementary material for: Beta-1,3 Oligoglucans Specifically Bind to Immune Receptor CD28 and May Enhance T Cell Activation
Source: Int J Mol Sci. 2021 Mar 18;22(6):3124. doi: 10.3390/ijms22063124 (PMC8003162; doi:10.3390/ijms22063124)
Supplement: Supplementary file 1 [file ijms-22-03124-s001.pdf]

Supplementary Information for

Beta-1,3 Oligoglucans Specifically Bind to Immune Receptor CD28 and May Enhance T Cell Activation

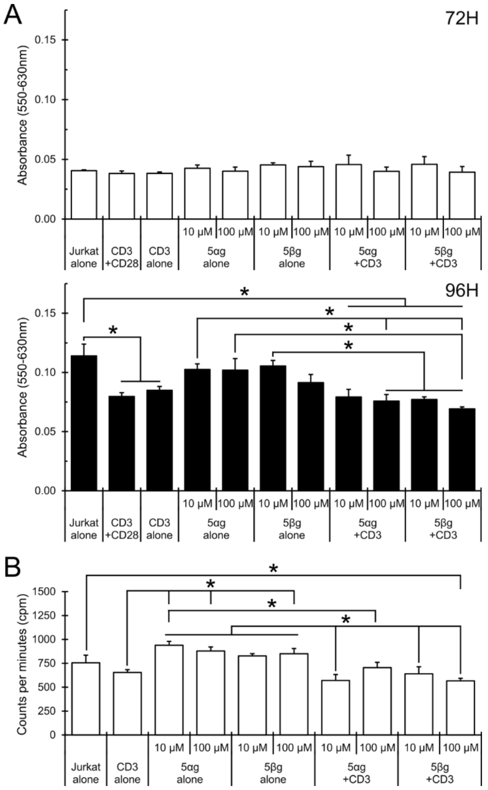

Figure S1: Neither 5-mer α-1,4 nor 5-mer β-1,3 oligoglucans altered cell proliferation significantly as demonstrated by an MTT assay **(A)** and a ^{3}H-thymidine incorporation assay **(B)**. Jurkat cells were treated with either α-1,4 or β-1,3 oligoglucans as indicated in the figure. The MTT assays were performed 72 hours (top) and 96 hours (bottom) after treatment. The ^{3}H-thymidine incorporation was determined during the 20–24 hours after the treatment.
